# A Novel Polyvinyl Alcohol/Salecan Composite Hydrogel Dressing with Tough, Biocompatible, and Antibacterial Properties for Infected Wound Healing

**DOI:** 10.3390/gels12010060

**Published:** 2026-01-08

**Authors:** Jiayu Li, Can Li, Qi Zhang, Zhenhao Rao, Qinghuan Meng, Miao Li, Juan Dai, Ke Deng, Pengfei Chen

**Affiliations:** 1 School of Food and Bioengineering, Xihua University, Chengdu 610039, China; 15700688805@163.com (J.L.); 18728653883@163.com (C.L.); 18481838481@163.com (Q.Z.); 15772128562@163.com (Z.R.); m18798294611@163.com (Q.M.); 18981910726@163.com (M.L.); 2 School of Laboratory Medicine, Chengdu Medical College, Chengdu 610500, China; daijuan@cmc.edu.cn

**Keywords:** Salecan, hydrogel dressing, interpenetrating network, infected wound

## Abstract

Polysaccharide-based wound dressings face challenges in mechanical properties and effective wound repair for infected wound surfaces. This study presents a novel polyvinyl alcohol (PVA)/Salecan (Sal) composite hydrogel dressing with high toughness, biocompatibility, and wound healing capabilities, developed using an interpenetrating polymer network strategy. The primary network was formed through electrostatic interactions between polydopamine (PDA) and biocompatible polysaccharide Salecan, followed by incorporation of AgNO_3_, which was in situ reduced to silver nanoparticles within the hydrogel. PVA was introduced as a secondary matrix, further reinforcing the hydrogel network through cyclic freeze–thawing. The resulting hydrogel exhibited a tensile strength of 0.31 MPa, an elongation at break of 158.9%, and a toughness of 31.16 J·m^−2^, demonstrating enhanced mechanical performance compared to both Salecan/PDA and previously reported Salecan/Fe^3+^ hydrogel. Co-culture experiments showed the hydrogel’s strong antibacterial effects, inhibiting 80.1% of *Escherichia coli* (*E. coli*) and 99.5% of *Staphylococcus aureus* (*S. aureus*). Fibroblast culture tests confirmed its excellent cytocompatibility. In vivo studies on infected wounds showed nearly complete healing in the *S. aureus* + hydrogel group within 12 days. Quantitative immunohistochemical analysis of CD31 revealed that hydrogel treatment significantly upregulated CD31 expression, indicating enhanced neovascularization. Complementary Western blot analysis further demonstrated that hydrogel-treated groups exhibited a marked downregulation of pro-inflammatory factors alongside CD31 upregulation. In summary, the PVA/Sal-based hydrogel represents a valuable strategy for reducing inflammation and promoting regeneration in the management of infected wounds.

## 1. Introduction

Skin tissue damage increases the risk of bacterial infection, leading to prolonged healing and severe tissue complications [1]. Wound dressings serve as temporary skin substitutes to accelerate healing and reduce pathological risks by protecting the wound site [2]. Traditional fibrous dressings often require frequent replacement due to their loose structures and inability to maintain a moist wound environment, which can lead to secondary injury [3]. Hydrogels are soft materials with good biocompatibility [4]. Typically, natural polysaccharide-based hydrogels have emerged as promising candidates due to their high water content, biocompatibility, biodegradability, and renewability [5].

Salecan is a novel water-soluble *β*-glucan purified from the extracellular polysaccharides of *Agrobacterium ZX09* and is an anionic polysaccharide [6,7]. All structural units in Salecan are connected by both α-(1→3) and β-(1→3) glycosidic linkages [8]. This distinctive molecular architecture endows Salecan with excellent biocompatibility, tunable rheological behavior, and versatile bioactive functions [9,10,11]. Additionally, Salecan can form highly viscous solutions at low concentrations and maintain stable viscosity over a wide range of temperatures and pH values. Moreover, the molecular chains of Salecan contain abundant carboxyl and hydroxyl groups, which facilitate chemical modification and cross-linking, demonstrating great promise for the development of tunable bioactive materials [12,13,14]. In our previous studies, biocompatible and injectable Salecan/Fe^3+^ hydrogels were developed, exhibiting excellent antimicrobial properties and a remarkable capacity to accelerate wound healing and tissue regeneration [15]. However, Salecan-based wound dressings still suffer from limited mechanical strength, which restricts their clinical usability. Although chemically crosslinked hydrogels offer improved mechanical properties, the presence of crosslinking agents is difficult to remove and would lead to cytotoxicity. Therefore, the development of a robust polysaccharide-based hydrogel dressing with good biocompatibility and antibacterial properties, capable of modulating wound inflammation and promoting tissue regeneration, is highly desirable.

Polyvinyl alcohol (PVA) is a water-soluble synthetic polymer that exhibits excellent hydrophilicity and high chemical stability, making it widely applicable in biomedical materials [16,17,18]. The application of PVA hydrogels in wound dressings is significantly constrained by their inherent bio-inertness and poor adhesion properties [19,20,21]. To address these limitations, blending PVA with natural polysaccharides has emerged as an effective strategy to enhance biocompatibility. For example, Pan et al. [22] developed a physically crosslinked hydrogel composed of PVA, human-like collagen, and sodium alginate. This formulation not only boosted biocompatibility but also promoted wound healing: wounds treated with it exhibited near-complete epithelialization within 10 days, alongside accelerated collagen fiber regeneration. However, this single-network physically crosslinked hydrogels typically demonstrate inferior mechanical performance when compared to their double-network counterparts [23,24].

**Figure 1:**
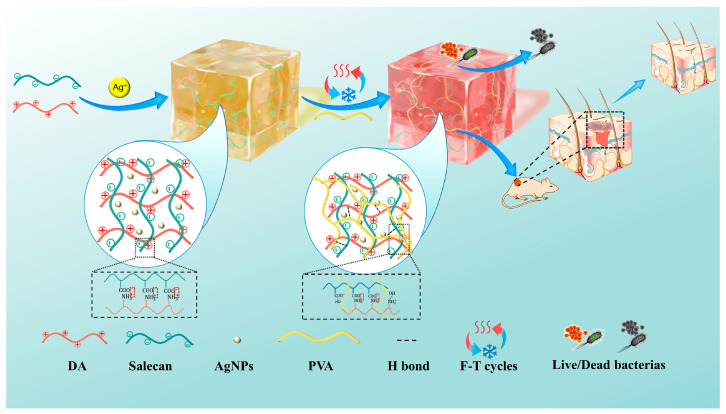
Schematic illustration of the fabrication process of polyvinyl alcohol/Salecan composite hydrogel and its application in infected wound healing.

In this work, an innovative polyvinyl alcohol (PVA)/Salecan (Sal) composite hydrogel dressing (Sal/DA/silver nanoparticles (AgNPs)/PVA) was developed, featuring high mechanical toughness, excellent biocompatibility, and significant wound healing potential, through an interpenetrating polymer network strategy based on physical crosslinking (Figure 1). Specifically, the first gel network was formed through electrostatic interactions between positively charged polydopamine and Salecan. The Ag^+^ ions incorporated into the initial network were reduced to silver nanoparticles in situ via the reducing capability of polydopamine, thereby enhancing both the mechanical strength and antibacterial activity of the hydrogel. Subsequently, PVA was introduced as a secondary matrix component and blended into the system, with the second gel network constructed through a freeze–thaw cycling process. This approach produced a composite antibacterial hydrogel featuring enhanced mechanical properties, which effectively addresses two key limitations: the insufficient mechanical strength of natural polysaccharide hydrogels and the biological inertness of PVA-based counterparts. The mechanical performance of the composite hydrogel is evaluated through rheological, tensile, and compressive testing. Its structural characteristics were examined by scanning electron microscopy (SEM). The antimicrobial efficacy of the prepared hydrogel was assessed using the colony count method, while its biocompatibility was evaluated through live/dead staining and Cell Counting Kit-8 (CCK-8) assay. Furthermore, the wound healing efficacy of the PVA/Sal-based hydrogel dressing is investigated in a rat model with circular full-thickness skin defects. The developed dual-network interpenetrating PVA/Sal-based composite hydrogel, integrating high toughness, antibacterial activity, and wound-healing promotion, represents a promising candidate for polysaccharide-based wound dressings and holds strong potential for future clinical applications in chronic wound care.

## 2. Results and Discussion

### 2.1. Synthesis of the Composite Hydrogel

**Figure 2:**
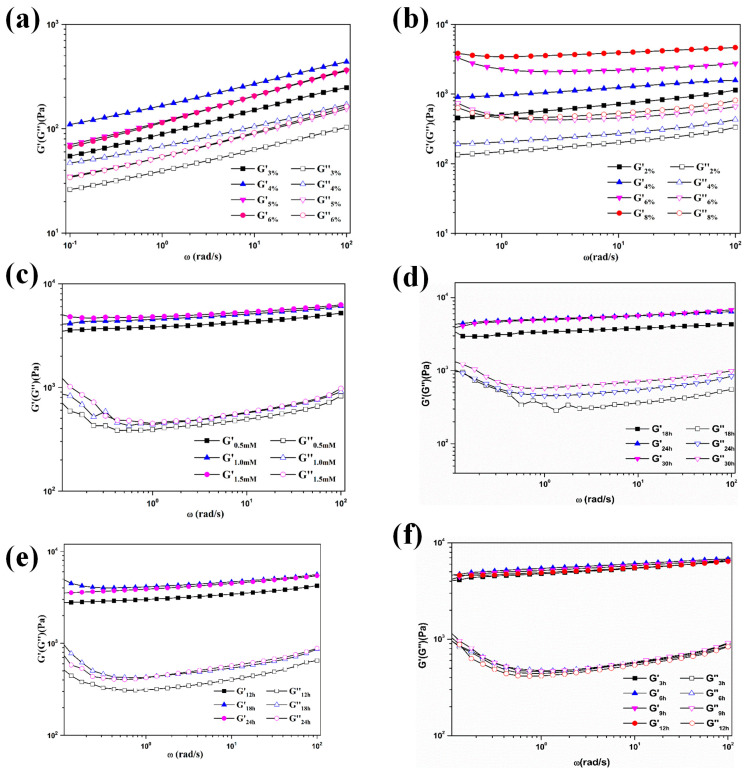
Effects of preparation conditions on rheological properties of hydrogels. (**a**–**c**) Oscillatory frequency sweep curves of Sal/DA hydrogels with different Salecan concentrations (**a**), Sal/DA/PVA hydrogels with different PVA concentrations (**b**), and Sal/DA/AgNPs /PVA hydrogels with different Ag^+^ concentrations (**c**); (**d**–**f**) Oscillatory frequency sweep curves of Sal/DA/AgNPs /PVA hydrogels with different freezing time (**d**), thawing temperature (**e**), and thawing time (**f**).

The effects of polymer and silver ion concentrations on rheological properties of the composite hydrogel were first investigated. Rheological measurements were conducted in dynamic mode, and amplitude sweep tests were performed to determine the linear viscoelastic region (LVR). Then, at a fixed shear strain of 1%, frequency sweeps (Figure 2a) revealed that shear storage modulus (G′) was greater than shear loss modulus (G″) across all Salecan concentrations, confirming the successful formation of a gel network between Salecan and PDA and demonstrating solid-like characteristics [25]. When the Salecan concentration exceeded 5%, the storage modulus of the hydrogel decreased. The high concentration (5%) of Salecan created steric hindrance, which restricted the stretching of polymer chains. Therefore, effective cross-linking between PDA and Salecan was prevented, which is similar to our previous study [15]. Although the hydrogel displayed good elasticity across the tested frequency range, G′ increased with frequency, suggesting an imperfect network structure [26]. PVA was incorporated to enhance the hydrogel network. As the PVA concentration increased, G′ showed a concentration-dependent enhancement (Figure 2b). Moreover, at a fixed PVA concentration, G′ remains nearly constant across different frequencies, indicating frequency independence and the formation of a stable network structure [27]. Furthermore, various concentrations of Ag^+^ were introduced into the hydrogel network, enhanced network strength with higher Ag^+^ content (Figure 2c). Considering the biocompatibility of the prepared composite hydrogel, the optimal formulation was determined to be 4% Salecan, 8% PVA, and 1.5 mmol·L^−1^ Ag^+^.

The freeze–thaw process, including freezing time, thawing temperature, thawing duration, and the freeze–thaw cycles, is a key factor in regulating the structure and functional properties of composite hydrogels (Figure 2d–f and Figure S1a). At a fixed shear strain of 0.05%, frequency sweep tests revealed that the composite hydrogel exhibited optimal viscoelastic properties when the freezing time was set to 18 h. Further extension of the freezing time did not significantly affect the viscoelastic properties of the hydrogel, suggesting that all microcrystalline crosslinking points in PVA had already been formed. Within the LVR, the composite hydrogel exhibited optimal viscoelasticity when thawed at 4 °C for 6 h. As the number of freeze–thaw cycles increased, the formation of microcrystalline crosslinking points also increased [28]. G′ was nearly identical between the fourth and fifth freeze–thaw cycles, suggesting that the microcrystalline crosslinking points in PVA reached saturation after four cycles, and no additional crosslinking could be induced under the same freeze–thaw conditions [29]. Therefore, the composite hydrogel was fabricated using the optimized parameters: freezing for 18 h, thawing at 4 °C for 6 h, and repeating the freeze–thaw cycle four times.

A specific concentration of AgNO_3_ solution was added dropwise to the hydrogel network under light-protected conditions to enhance its antibacterial and mechanical properties. Silver ions gained electrons from the catechol groups in PDA and were reduced to AgNPs. The order of AgNO_3_ addition had little effect on the elasticity of the hydrogel (Figure S1b). However, the cumulative release of Ag^+^ varied significantly depending on the sequence of AgNO_3_ addition (Figure S1c,d). For Sal/DA/AgNPs/ PVA, a greater amount of Ag^+^ was reduced to AgNPs when AgNO_3_ solution was added after the formation of PDA. The subsequent addition of PVA effectively dispersed the AgNPs [30], leading to the formation of smaller nanoparticles that facilitated their release from the composite hydrogel. Thus, Ag^+^ were continuously released from the Sal/DA/PVA/AgNPs composite hydrogel, with the cumulative release increasing over time, and the cumulative release of silver reached 50% after 48 h under pH = 2. Therefore, Ag^+^ was incorporated into the first layer of the hydrogel network to ensure its higher antibacterial performance.

Both the G′ and G″ of the composite hydrogel were significantly higher than those of the single-component hydrogels prior to cyclic freeze–thaw treatment (Figure S1e,f). The incorporation of PDA and AgNPs significantly increased the entanglement density of molecular chains in the system. Moreover, the uniformly dispersed AgNPs acted as effective crosslinking points, further enhancing the elastic response of the material. Notably, the G′ of both hydrogels increased with frequency, indicating dynamic relaxation behavior and incomplete crosslinking within their 3D network structures. After cyclic freeze–thaw treatment, the composite hydrogel maintained a significant advantage in both G′ and G″ over the single-component hydrogels and exhibited a more pronounced increasing trend. The synergistic interaction between PDA and AgNPs in the composite hydrogel facilitated the formation of a more robust and densely crosslinked network structure. Additionally, this composite crosslinking system demonstrated excellent resistance to freeze-induced damage, effectively preventing structural degradation under low-temperature conditions.

### 2.2. Structural and FTIR Characterization

**Figure 3:**
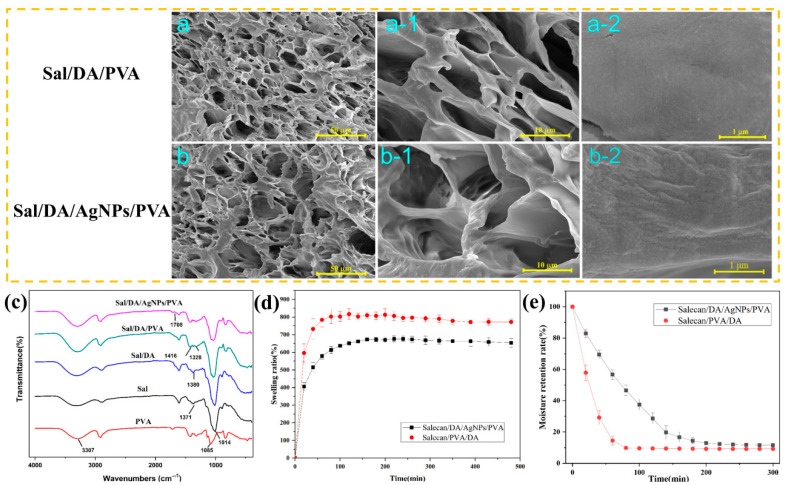
Structural and performance characterization of composite hydrogels. (**a**,**b**) Cross-sectional SEM images of Sal/DA/PVA (**a**) and Sal/DA/AgNPs/PVA (**b**), (**a-1**), (**a-2**), (**b-1**), and (**b-2**) are enlarged views of the cross sections in (**a**) and (**b**), respectively; (**c**) FTIR of hydrogels; (**d**) swelling behaviors of hydrogels; (**e**) water retention capacity of hydrogels.

As shown in SEM images, all composite hydrogels exhibited a uniformly distributed and highly interconnected porous structure (Figure 3a,b). The porous structure mainly originates from the microcrystalline crosslinking of PVA molecules induced during the freezing, coupled with the physical entanglement of Salecan chains. Notably, compared to the Sal/DA/PVA composite hydrogel, the Sal/DA/AgNPs/PVA composite hydrogel exhibited larger pores sizes and more interconnected channels, which can be attributed to the reduction of Ag^+^ into AgNPs by PDA [31]. The reduction of Ag^+^ to AgNPs by the residual NH_2_ and phenol groups in polydopamine broke the hydrogen bonds between polydopamine and Salecan. This disruption resulted in a hydrogel with a larger pore size. The porous structure facilitates the absorption of wound exudate by the dressing while maintaining excellent breathability. Moreover, the rougher surface of pores in the Sal/DA/AgNPs/PVA composite hydrogel, also indicates the successful incorporation of AgNPs (Figure 3(b-2)).

As shown in Figure 3c, FTIR spectroscopy results revealed the characteristic peaks of Salecan and PVA. Peaks at 897 cm^−1^ and 836 cm^−1^ were both attenuated after blending Salecan with PVA, indicating molecular interactions between PVA and Sal/DA hydrogels that modified their crystalline structures [32]. After the addition of AgNO_3_, a new peak appeared at 1708 cm^−1^, corresponding to the -C=O stretching vibration, which directly confirms the successful reduction of Ag^+^ to AgNPs by PDA [33]. The bending vibration at 1092 cm^−1^ exhibited a redshift to 1034 cm^−1^, attributed to hydrogen bonding between PVA and Sal/DA hydrogels [34], thereby enhancing the structural integrity of the hydrogel.

Water absorption approached saturation within approximately 2 h, reaching a final swelling ratio of 651.09% (Figure 3d). The excellent water absorption capacity of the hydrated hydrogel is attributed to three-dimensional network structure and the abundance of hydroxyl groups in Salecan and PVA, both enhancing the efficient absorption of wound exudate. Hydrophilic chains within the hydrogels serve as water-release channels upon network collapse [35], facilitating the efficient release of water from the gel matrix. This mechanism prevents fluid accumulation while maintaining a moist wound environment conducive to accelerated healing. However, the increased crosslinking density by AgNPs reduces swelling performance of Sal/DA/AgNPs/PVA hydrogel. The water in Sal/DA/AgNPs/PVA composite hydrogel released approximately 88.4% within 6 h (Figure 3e), attributed to the hydrophilic polymer chains and porous structures. The uniform distribution of AgNPs in the Sal/DA/AgNPs/PVA composite hydrogel helps to partially mitigate rapid water loss [36], resulting in slower water loss compared with Sal/DA/PVA. Overall, the prepared composite hydrogel demonstrates ideal wound dressing characteristics, effectively absorbing exudate while maintaining an optimal wound microenvironment.

### 2.3. Adhesive and Mechanical Properties

**Figure 4:**
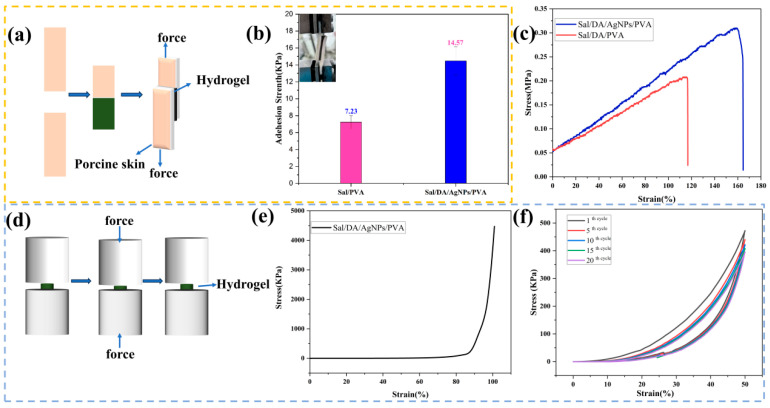
Mechanical properties of hydrogels. (**a**,**b**) Adhesive capability of the composite hydrogel; (**c**) stretching stress–strain curves; (**d**) schematic illustration of the compression test; (**e**,**f**) compression curves, (**e**) and cyclic compression curves (**f**) of hydrogels.

To meet the clinical requirements for chronic wound care, hydrogel dressings should effectively adhere to wound sites, thereby preventing bacterial colonization and fluid leakage [37]. The adhesive performance of the hydrogel was assessed on fresh porcine skin, which served as a substrate to simulate human tissue (Figure 4a,b). The Sal/DA/AgNPs/PVA composite hydrogel exhibited approximately twice the adhesive strength of the Sal/PVA hydrogel. This enhancement is attributed to PDA, which forms multiple hydrogen bonds with the skin surface, thereby improving adhesion. Moreover, the increased contact area also contributes to the improved adhesion. In situ reduction of AgNPs by PDA increases the surface roughness of the hydrogel, thereby significantly enhancing the contact area.

The prepared hydrogels exhibited excellent tensile and compressive properties (Figure 4c). The Sal/DA/PVA composite hydrogel exhibited a tensile stress of 0.21 MPa, a fracture elongation of 117.1%, and a fracture energy of 15.5 J·m^−2^. Following in situ synthesis of AgNPs, the Sal/DA/AgNPs/PVA composite hydrogel demonstrated enhanced mechanical properties, with stress increased to 0.31 MPa, fracture elongation to 158.9%, and fracture energy to 31.16 J·m^−2^, confirming its capability to withstand substantial deformation and conform well to wound surfaces. The composite hydrogel exhibited ultra-high strength and ductility (Figure 4e). The Sal/DA/AgNPs/PVA hydrogel possessed excellent elastic shape recovery under stress compression (Figure 4f). The non-overlapping loading and unloading curves form a minimal hysteresis loop, which is a hallmark of viscoelasticity, indicating little energy dissipation during compression.

### 2.4. In Vitro Antibacterial Properties

**Figure 5:**
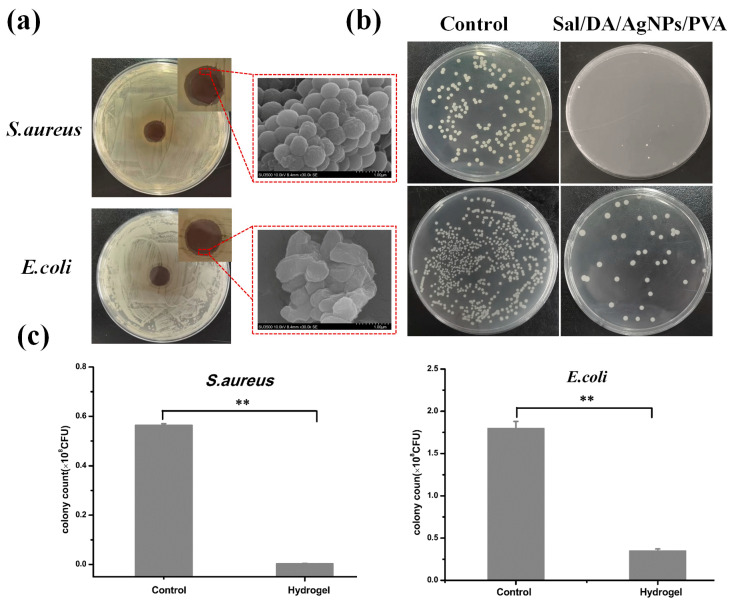
Antibacterial activity of hydrogels against *E. coli* and *S. aureus*. (**a**) Antibacterial circle and SEM; (**b**) diluted coating plate; (**c**) corresponding statistical data of *S. aureus* and *E. coli* colonies, where ** *p* < 0.01 (*n* = 3).

Bacterial infection is a common complication in wound healing. Inhibition zone and SEM analyses confirmed the antibacterial efficacy of the hydrogel (Figure 5a). Distinct inhibition zones were observed on both *S. aureus* and *E. coli* culture plates, indicating that the Sal/DA/AgNPs/PVA composite hydrogel not only prevents bacterial adhesion on its surface but also effectively inhibits bacterial proliferation in the surrounding environment. The composite hydrogel exhibited a stronger inhibitory effect on *S. aureus* than on *E. coli* (Figure 5b), which can be attributed to the differing antibacterial mechanisms of AgNPs and negative Salecan polymer against Gram-positive and Gram-negative bacteria [15,38]. The inhibition rates of Sal/DA/AgNPs/PVA composite hydrogel against *S. aureus* and *E. coli* were 99.5% and 80.1%, respectively (Figure 5c). This excellent antibacterial performance can be attributed to the release of silver ions into the solution or the direct contact of Ag released from AgNPs with bacterial cells, leading to ion-mediated bacteria inactivation [39].

### 2.5. In Vitro Biocompatibility

**Figure 6:**
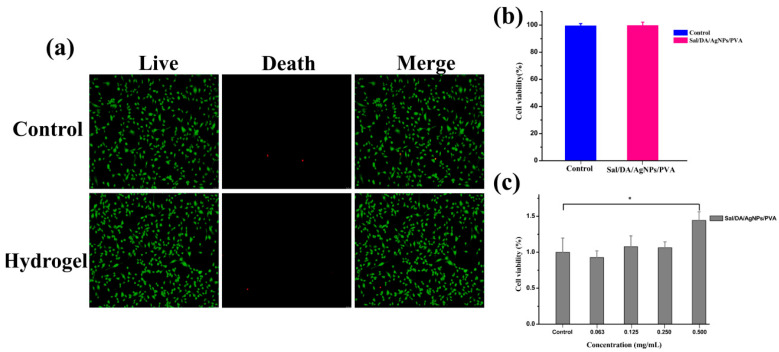
(**a**) Fluorescent images of live/dead staining; (**b**) statistical data from the live/dead assay; (**c**) statistical data from the CCK-8 assay. * *p* < 0.05 and *n* = 3.

The hydrogels exhibit excellent cytocompatibility with cells. The biocompatibility of the composite hydrogel was assessed using live/dead staining and the CCK-8 assay. The viability of human skin fibroblast (HSF) cells cultured with Sal/DA/AgNPs/PVA composite hydrogel was 99.8%, which was nearly identical to that of the control group (99.5%) (Figure 6a,b). The results indicate that the composite hydrogel exhibited no cytotoxicity. The CCK-8 assay was used to evaluate the proliferation ability of HSF cells on composite hydrogels (Figure 6c). The cell growth rate under low concentrations of Sal/DA/AgNPs/PVA composite hydrogels was nearly identical to that of the control group, further confirming that the composite hydrogels had no inhibitory effect on cell proliferation. The number of HSF cells increased significantly when the hydrogel concentration was increased to 0.5 mg/mL, suggesting that the polysaccharide-based hydrogel promotes HSF cell proliferation [40] and may therefore accelerate wound healing. In vitro scratch assays also confirm that the composite hydrogel promotes cell migration (Figure S2).

### 2.6. In Vivo Evaluation of Wound Healing

**Figure 7:**
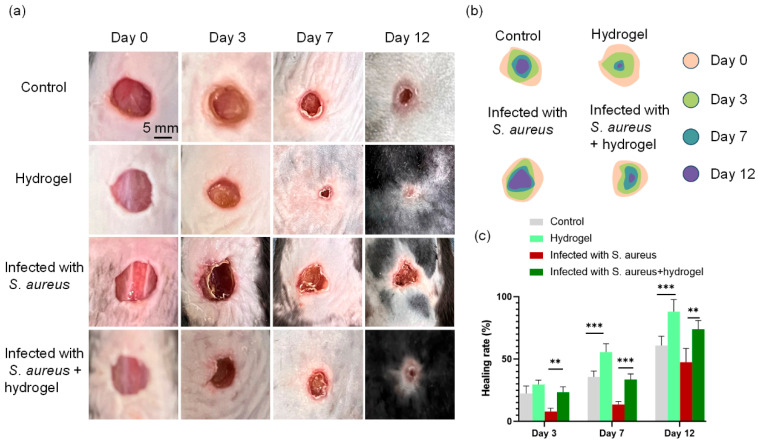
In vivo infected wound healing evaluation. (**a**) Wound photographs in various treatment groups at different time points; (**b**) schematic illustration of wound closure over time; (**c**) quantitative analysis of wound healing rate at different time points. ** *p* < 0.01 and *** *p* < 0.001, *n* = 12.

*S. aureus* is one of the most prevalent pathogens in clinical skin and wound infections, including surgical site infections, burn wounds, and diabetic foot ulcers. To evaluate the infected wound healing efficacy of Sal/DA/AgNPs/PVA composite hydrogel in vivo, a full-thickness skin defect model (diameter 10 mm) and an *S. aureus*-infected wound model were established on the dorsum of rats. The rats were divided into four groups: (1) control, (2) hydrogel, (3) infected with *S. aureus*, and (4) infected with *S. aureus* + hydrogel. The hydrogel-treated group exhibits significantly enhanced healing compared to untreated controls (Figure 7). By day 12, wounds treated with the hydrogel exhibited nearly complete closure, with a healing rate of about 88.07%, whereas wounds in the control group remained largely unhealed (60.97%). A similar trend was observed under infected wounds groups, where infected wounds in the hydrogel-treated group showed substantial recovery (73.93%), in contrast to the markedly impaired healing in the *S. aureus* group (47.42%). The application of the hydrogel markedly enhanced the wound healing process compared to the untreated control group. The healing efficacy of infected wounds was lower than that observed in the *S. aureus* + hydrogel group.

**Figure 8:**
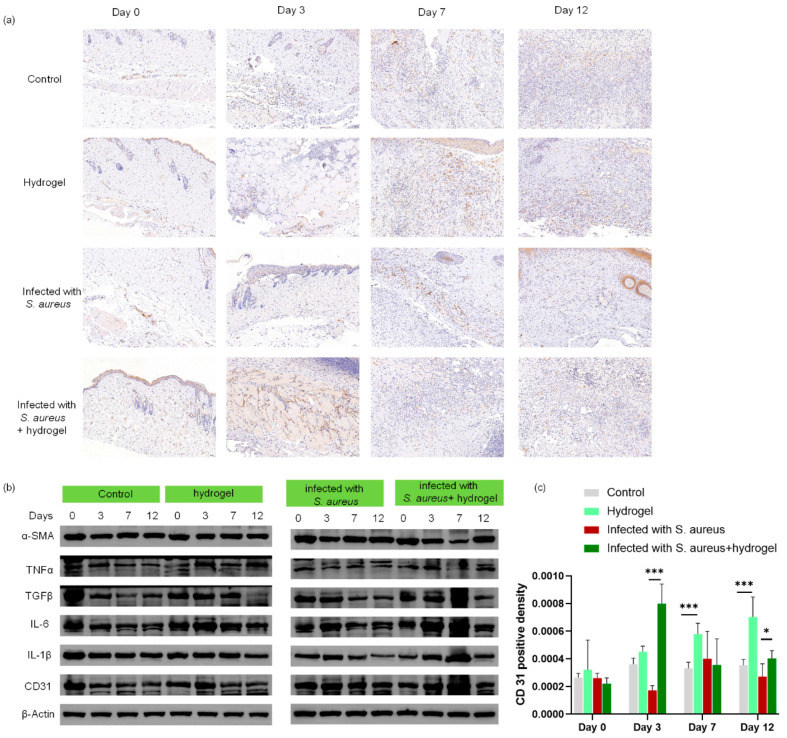
Histological and WB analysis of wound tissues in rats. (**a**) CD31 immunohistochemical staining of wound tissue at different time points, and (**c**) quantitative analysis; (**b**) WB analysis of differential expression of *α*-smooth muscle actin (α-SMA), tumor necrosis factor-α (TNF-α), transforming growth factor-*β* (TGFβ), interleukin-6 (IL-6), interleukin-1β (IL-1β), and CD31 of wounds at different time points. * *p* < 0.05, *** *p* < 0.001, and *n* = 12.

Immunohistochemical staining and Western blotting (WB) assay were performed on rat tissues to confirm the healing efficacy of the hydrogel. Angiogenesis is a critical process in wound healing, and platelet endothelial cell adhesion molecule-1 (CD31) serves as a well-recognized marker of neovascularization [41,42]. Immunohistochemical staining and quantitative analysis of CD31 revealed that hydrogel treatment markedly enhanced CD31 expression (Figure 8a,c), indicating a significant promotion of new blood vessel formation. WB analysis demonstrated an obvious decrease in inflammatory factor expression accompanied by an upregulation of CD31 in the hydrogel-treated groups (Figure 8b). These findings demonstrate that hydrogel treatment significantly accelerates wound closure compared to untreated controls, even under bacterial infection.

**Figure 9:**
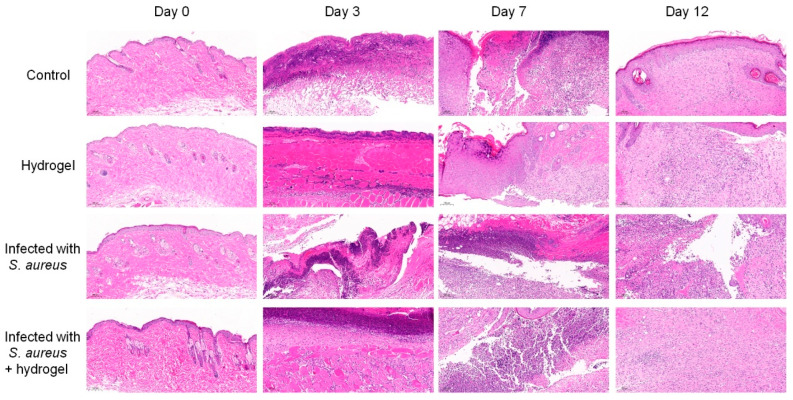
Representative hematoxylin and eosin (H&E) staining during the wound healing process.

Histological evaluation of wound healing using H&E-stained sections confirmed the therapeutic efficacy of the composite hydrogel (Figure 9). On day 3 post-operation, all groups exhibited significant infiltration of inflammatory cells. From day 6 onward, the hydrogel-treated groups (including hydrogel and *S. aureus* + hydrogel groups) exhibited a significant reduction in inflammatory cells and the onset of vascular regeneration, which can be attributed to the sustained antibacterial activity of AgNPs that prevented infection-induced inflammation. By day 12, the hydrogel-treated normal wounds and infected wounds all demonstrated nearly complete dermal regeneration, characterized by a significant increase in hair follicles and blood vessels, thereby confirming its efficacy in promoting chronic wound healing. In vivo results confirm that this novel hydrogel effectively alleviates inflammation and promotes tissue regeneration within 12 days in an infected rat wound model.

## 3. Conclusions

In summary, a tough, biocompatible, and antibacterial PVA/Sal-based hydrogel dressing was successfully developed to promote infected wound healing. Microcrystalline cross-linking points and the entangled molecular chains of PVA form porous structures, enhancing the mechanical properties of the hydrogel. The hydrophilic molecular chains and porous structures endow the hydrogels with significant water retention and swelling properties. Hydrogel dressing also exhibits a strong adhesive capability due to the introduction of PDA. Ag^+^ ions are in situ reduced to AgNPs within the hydrogel network by PDA, enhancing its antibacterial and mechanical properties, including good compressive resistance and self-recovery ability. In vitro antibacterial tests demonstrated that the composite hydrogel exhibited significant inhibitory effects against common pathogenic bacteria, with inhibition rates of 99.5% against *S. aureus* and 80.1% against *E. coli*. In vitro cell culture experiments showed that the composite hydrogel exhibited no cytotoxicity and demonstrated significant cytocompatibility. The infected wounds treated with hydrogel achieved near-complete wound closure with minimal scarring within 12 days. The results offer a valuable strategy for designing tough, adhesive, antibacterial, and biocompatible polysaccharide-based hydrogel dressings to promote infected wound healing and hold strong potential for future clinical applications in chronic wound care.

## 4. Materials and Methods

### 4.1. Materials

Salecan (purity: 95%) was obtained from Sichuan Synlight Biotech Ltd. (Deyang, China). Dopamine, PVA, and AgNO_3_ were purchased from Shanghai Aladdin Biotech Ltd. (Shanghai, China). All experiments utilized deionized water (18.2 MΩ·cm at 25 °C), which was obtained from a Milli-Q Plus water purification system. The chemicals utilized in this experiment were analytical grade and did not undergo additional purification.

### 4.2. Preparation of the Composite Hydrogel

The total system volume for preparation of the hydrogel was 20 mL, with the concentration of DA fixed at 2 mg/mL [43]. Firstly, a DA solution was prepared from a Tris-HCl buffer solution (pH 8.5). The DA solution was gradually added dropwise to the Salecan solution (4%, *w*/*v*) under continuous stirring. The first layer of gel network, namely Sal/DA hydrogel, was formed through the electrostatic interaction between DA and Salecan. Subsequently, 1.5 M AgNO_3_ solution was added to this initial hydrogel network. Then, the PVA solution (8%, *w*/*v*) was thoroughly mixed with the above system. Subsequently, a second gel network was established through a cyclic freeze–thaw process, with four cycles of freezing (−20 °C, 18 h) and thawing (4 °C, 6 h). The composite hydrogel was obtained after the cyclic freeze–thaw process.

### 4.3. Fourier-Transform Infrared (FTIR) Spectroscopy

Dried hydrogel was obtained through freeze-drying. The samples for testing were prepared using the KBr tablet method. Chemical groups of Salecan polymer, Sal/DA hydrogel, and Sal/DA/AgNPs/PVA hydrogel were characterized by FTIR spectroscopy (PerkinElmer UATR Two, Yokohama, Japan).

### 4.4. Scanning Electron Microscopy (SEM)

The cross-sectional structures of the composite hydrogels were observed by SEM (Thermo Scientific Apreo 2C, Waltham, MA, USA). The hydrogels were fractured in liquid nitrogen and then coated with a thin layer of Au before the SEM test.

### 4.5. Dynamic Rheological Characterization

The rheological properties were measured with an rotational rheometer (Anton Paar CR302, Graz, Austria) fitted with 25 mm parallel plates. Initially, amplitude sweep tests were performed to determine the linear viscoelastic region (LVR). Based on this, frequency sweep tests were then carried out at a constant strain of 1% (within the LVR), covering an angular frequency range from 0.1 to 100 rad/s. All experiments were maintained at a plate gap of 1 mm and a temperature of 25 °C. The curves of shear storage modulus (G′) and shear loss modulus (G″) were recorded.

### 4.6. Water Retention Capability

The initial mass of the hydrogel was recorded as *M*_0_. Subsequently, the hydrogel was put in a 37 °C air oven. At certain time intervals, the mass of hydrogel was weighed as *M*_i_ [44]. The water retention capacity was calculated by the following formula:

(1) $$\mathrm{Water}\;\mathrm{retention}\;=\;1\;-\;\frac{M_{0}-M_{i}}{M_{0}}\;\times \;\mathrm{100}\%$$

### 4.7. Swelling Performance

The swelling capacity of composite hydrogel samples at different time points was determined by a gravimetric method [45,46]. The hydrogels were weighed, and their initial mass was recorded as *W*_0_. Subsequently, the hydrogels were fully immersed in PBS (pH 7.4). At predetermined time intervals, the hydrogels were taken out, and their mass was recorded as *W*_i_. Prior to weighing, surface moisture was carefully blotted using filter paper. The swelling ratio was calculated using the following formula:

(2) $$\mathrm{Swelling}\;\mathrm{ratio}\;=\;\frac{W_{i}-W_{0}}{W_{0}}\;\times \;\mathrm{100}\%$$

The adhesive strength of the hydrogels was quantified via shear tests, using porcine skin as the substrate. Following pretreatment to remove hair and excess oil, uniform-thickness fresh pig skin was cut into strips and soaked in 37 °C PBS. A 1 cm^2^ hydrogel patch was applied to one end of a strip, after which two strips were horizontally overlapped at the treated ends and pressed together. After a set bonding time, the sample was fixed at both ends to the clamps of a universal testing machine for mechanical evaluation. After the sample was incubated at 37 °C for 3 h, the adhesion strength was measured at a speed of 5 mm.min^−1^ [19]. The adhesive strength was calculated using the following formula:

(3) $$\mathrm{Adhesive}\;\mathrm{strength}\;=\;\frac{F}{S}$$

where *F* represents the maximum force recorded during the tensile process, and *S* denotes the contact area between two pieces of pig skin.

### 4.8. Mechanics Performance Test

The mechanical properties of the composite hydrogels were tested by a universal stretching machine [47]. The sample was prepared using a dumbbell-shaped mold (length × width × height: 50 × 8 × 2 mm) and subjected to tensile testing at a strain rate of 10 mm/min. The elastic modulus E was determined from the slope of the stress (σ)—strain (ε) curve, while the fracture energy was calculated as the area under the stress–strain curve.

Elongation at break was calculated using the following formula:

(4) $$\mathrm{Elongation}\;\mathrm{at}\;\mathrm{break}\;=\;\frac{L_{1}-L_{0}}{L_{0}}\;\times \;\mathrm{100}\%$$

where *L*_1_ represents the distance between the fracture surfaces after the sample breaks, and *L*_0_ represents the original gauge length of the sample.

The compression properties of the composite hydrogel were tested using an electronic universal testing machine. First, the hydrogel was prepared into a cylindrical sample with a diameter of 16 mm and a height of 10 mm. A single-cycle compression performance test was carried out at room temperature. The hydrogel was compressed at a rate of 5 mm/min until rupture occurred. The test was completed, and the compressive stress–strain curve was obtained. The sample was then subjected to cyclic compression testing at 60% strain and a rate of 2 mm/min, followed by recovery to 0% strain at the same rate. The loading and unloading cycles were repeated 20 times, and the corresponding compressive stress–strain curves were recorded.

### 4.9. In Vitro Antibacterial Activity

*E. coli* and *S. aureus* were used to evaluate the antibacterial activity of the composite hydrogels based on the inhibition zone method and spread plate method [48]. First, the bacteria were inoculated into the culture medium for subculturing to determine their concentration. Next, *E. coli* and *S. aureus* suspensions at a concentration of 2 × 10^8^ CFU/mL were evenly spread onto the surface of a solidified agar plate, and a composite hydrogel (10 × 10 mm) was placed onto the agar surface. Finally, the agar plates were incubated at 37 °C for 18 h. Then they were photographed, and bacterial colonies were counted.

The bacteriostatic rates were further evaluated. 1 g of the composite hydrogel was added to the bacterial suspension at a concentration of 10^6^ colony-forming units (CFU)/mL and incubated for 24 h. A bacterial suspension without the composite hydrogels served as the blank control. Then, 100 μL of each mixture was evenly spread onto the surface of solidified agar plates. Finally, the agar plates were incubated at 37 °C for 18 h, after which they were photographed, and bacterial colonies were counted.

The antibacterial rate was calculated using the following formula:

(5) $$\mathrm{Inhibition}\;\mathrm{rate}\;(\%)=\frac{{CFU}_{0}-{CFU}_{1}}{{CFU}_{0}}\;\times \;100\%$$

where *CFU*_1_ represents the number of colony-forming units (CFUs) in the presence of the composite hydrogel, and *CFU*_0_ represents the number of bacterial units in the control group.

### 4.10. Ag^+^ Release Profile

The prepared composite hydrogels and non-silver-loaded hydrogels were immersed in 20 mL PBS at pH 2 and pH 5.4, as previously described. The mixture was incubated in a shaker at 37 °C and 40 rpm. At regular intervals, 2 mL of solution was withdrawn and replaced with an equal volume of fresh PBS. A 0.5 mL aliquot of the 2 mL release solution was taken for microwave digestion. After pre-digestion at 120 °C for 60 min, programmed digestion was performed under the following conditions: 120 °C for 2 min, 150 °C for 5 min, and 185 °C for 50 min. Following digestion, the acid was evaporated on an electric heating plate at 120 °C. Once the volume was reduced to approximately 1 mL, the solution was diluted to a final volume of 10 mL with 2% (*w*/*v*) nitric acid.

Following microwave digestion, the concentration of Ag^+^ was determined by inductively coupled plasma–atomic emission spectroscopy (ICP-AES; Avio 200, PerkinElmer, Shelton, CT, USA) [49]. Prior to the test, standard solutions with varying concentrations of silver were prepared to establish a standard curve. Subsequently, the concentration of Ag^+^ in the samples was measured at each time point to calculate the cumulative silver release from Sal/DA/PVA/AgNPs composite gel.

The cumulative release was calculated using the following formula:

(6) $$\mathrm{Cumulative}\;\mathrm{release}\;(\%)=\frac{V_{0}C_{n}+V_{e}\sum_{1}^{n-1}C_{i-1}}{m}\;\times \;100\%$$

where *V*_0_ represents the total volume of the releasing medium, *V*_e_ denotes the displacement volume, *C*_n_ indicates the concentration of release solution at the final time point, and m denotes the total silver content in the hydrogel.

### 4.11. Cytocompatibility of the Composite Hydrogel

Human skin fibroblasts (HSFs) were purchased from Jiangsu Yaohai Biotechnology Co., Ltd. (Taizhou, China). In total, 1 mL of frozen cell suspension was thawed in a water bath at 37 °C, mixed with 4 mL of medium (Dulbecco’s Modified Eagle Medium (DMEM) supplemented with 10% fetal bovine serum and 1% penicillin/streptomycin), and centrifuged at 500 G rotation speed for 5 min. After removing the supernatant, 2 mL of fresh medium was added, the cells were gently resuspended, and then transferred to a culture bottle for overnight incubation.

The cytocompatibility of the composite hydrogel was investigated via live/dead assay [50] and CCK-8 assay [40]. Cell activity tests are performed as follows: after removing PBS, 500 μL trypsin was added for digestion. Subsequently, 2 mL medium was added to neutralize the trypsin and mix the cells. After transferring the cell suspension to a sterile tube, it was centrifuged (500 G, 5 min), and the supernatant was discarded. The cells were resuspended at 40,000 cells/mL, and 50 μL aliquots were dispensed into a 96-well plate. Hydrogel solutions of different concentrations were then added to the wells. At this time, the cell density in each well was 2000 cells/mL. The 96-well plates were transferred to the cell incubator and incubated for 24 h. After incubation, each well was rinsed with 100 mL PBS, and the wash solution was removed. Then, CCK-8 reagent was added, and the plate was incubated for 1 h. Following this, wells were washed three times with PBS. Finally, 100 μL DMSO was added to each well, and the plate was shaken for 3 min before measuring absorbance at 450 nm. The absorbance of the 96-well plate was read using an enzyme reader. Cell viability was determined using a Calcein-AM/PI double-staining assay. Cell viability was then observed under an inverted fluorescence microscope.

### 4.12. In Vivo Infected Wound-Healing Model

Sprague–Dawley rats were used in the in vivo experiment. The promoting wound healing abilities of the hydrogels were investigated in a full-thickness rat skin defect model and a full-thickness rat skin defect model infected with *S. aureus*. The animal experiments were approved by the Chengdu Medical College Experimental Animal Ethics Committee. The rats were anesthetized with 3% isoflurane. After hair removal, a full-thickness wound (diameter = 10 mm) on the dorsum of each rat was punched with a 10 mm biopsy device. Afterwards, 48 male Sprague–Dawley rats weighing about 250 g were randomly divided into four groups: control, hydrogel, infected with *S. aureus*, and infected with *S. aureus* + hydrogel. The treatments administered were as follows: the control group received only the wound creation. The hydrogel group had wounds covered with the composite hydrogel dressing. To establish a chronic infection model, wounds in the infected group were inoculated with 200 μL of *S. aureus* (strain Wichita, ATCC 29213) at a concentration of 1 × 10^6^ CFU/mL [51]. The infected + hydrogel group received the same bacterial inoculation, followed immediately by the application of the composite hydrogel dressing. Under standard conditions, all rats were housed in individually ventilated cages and maintained on a normal diet. Wound dressings were changed every three days, and the wound areas were documented through photography and measurement to allow for calculation of the healing rate using the formula:

(7) $$\mathrm{Wound}\;\mathrm{healing}\;\mathrm{rate}\;(\%)\;=\frac{S_{0}-S}{S_{0}}\;\times \;100\%$$

where *S* is the wound area in the healing process, and *S*_0_ is the wound area on the day the wound was created.

The wound tissues were collected on days 0, 3, 7, and 12, and frozen at −80 °C for WB analysis. The harvested wound tissues on days 0, 3, 7, and 12 were fixed with paraformaldehyde for H&E staining and CD31 immunohistochemical staining, following the standard protocols [52]. Histological sections were recorded with a digital microtome scanner and analyzed with ImageJ (version 1.54p).

### 4.13. Statistical Analysis

Statistical analyses were conducted using SPSS version 17.0 software by applying a *t*-test to examine the statistical differences between the mean values. Data are expressed as the mean ± standard deviation. Statistical significance was assessed with the threshold set at * *p* < 0.05 (*n* = 3).

## Data Availability

The original contributions presented in this study are included in the article. Further inquiries can be directed to the corresponding authors.

## Supplementary Materials

The following supporting information can be downloaded at: https://www.mdpi.com/article/10.3390/gels12010060/s1, Figure S1. (a) Oscillatory frequency sweep curves of Sal/DA/AgNPs/PVA hydrogels with different number of freeze-thaw cycles; (b) Oscillatory frequency sweep curves under different Ag^+^ addition sequences; (c,d) Ag^+^ release profiles at pH 2.0 (c) and pH 5.4 (d); (e,f) Oscillatory frequency sweep curves of Sal/PVA and Sal/DA/AgNPs/PVA before (e) and after (f) freeze-thaw cycling. Figure S2. In vitro scratch assays to evaluate the effect of the Sal/DA/AgNPs/PVA composite hydrogel on cell migration.
