# Supplementary material for: A Novel Polyvinyl Alcohol/Salecan Composite Hydrogel Dressing with Tough, Biocompatible, and Antibacterial Properties for Infected Wound Healing
Source: Gels. 2026 Jan 8;12(1):60. doi: 10.3390/gels12010060 (PMC12840809; doi:10.3390/gels12010060)
Supplement: Supplementary file 1 [file gels-12-00060-s001.zip › gels-4079382-supplementary.pdf]

Supporting Information

A Novel Polyvinyl Alcohol/Salecan Composite Hydrogel Dressing with Tough, Biocompatible, and Antibacterial Properties for Infected Wound Healing

Jiayu Li[1], Can Li[1], Qi Zhang[1], Zhenhao Rao[1], Qinghuan Meng[1], Miao Li[1], Juan Dai[2], Ke Deng[1,*] and Pengfei Chen[1,*]

[1] School of Food and Bioengineering, Xihua University, Chengdu 610039, China; 15700688805@163.com (J.L.); 18728653883@163.com (C.L.); 18481838481@163.com (Q.Z.); 15772128562@163.com (Z.R.); m18798294611@163.com (Q.M.); 18981910726@163.com (M.L.)

[2] School of Laboratory Medicine, Chengdu Medical College, Chengdu 610500, China; daijuan@cmc.edu.cn

* Correspondence: kelvin_de@126.com (K.D.); pengfeimj1028@163.com (P.C.)

Supplementary Figures S1 and S2

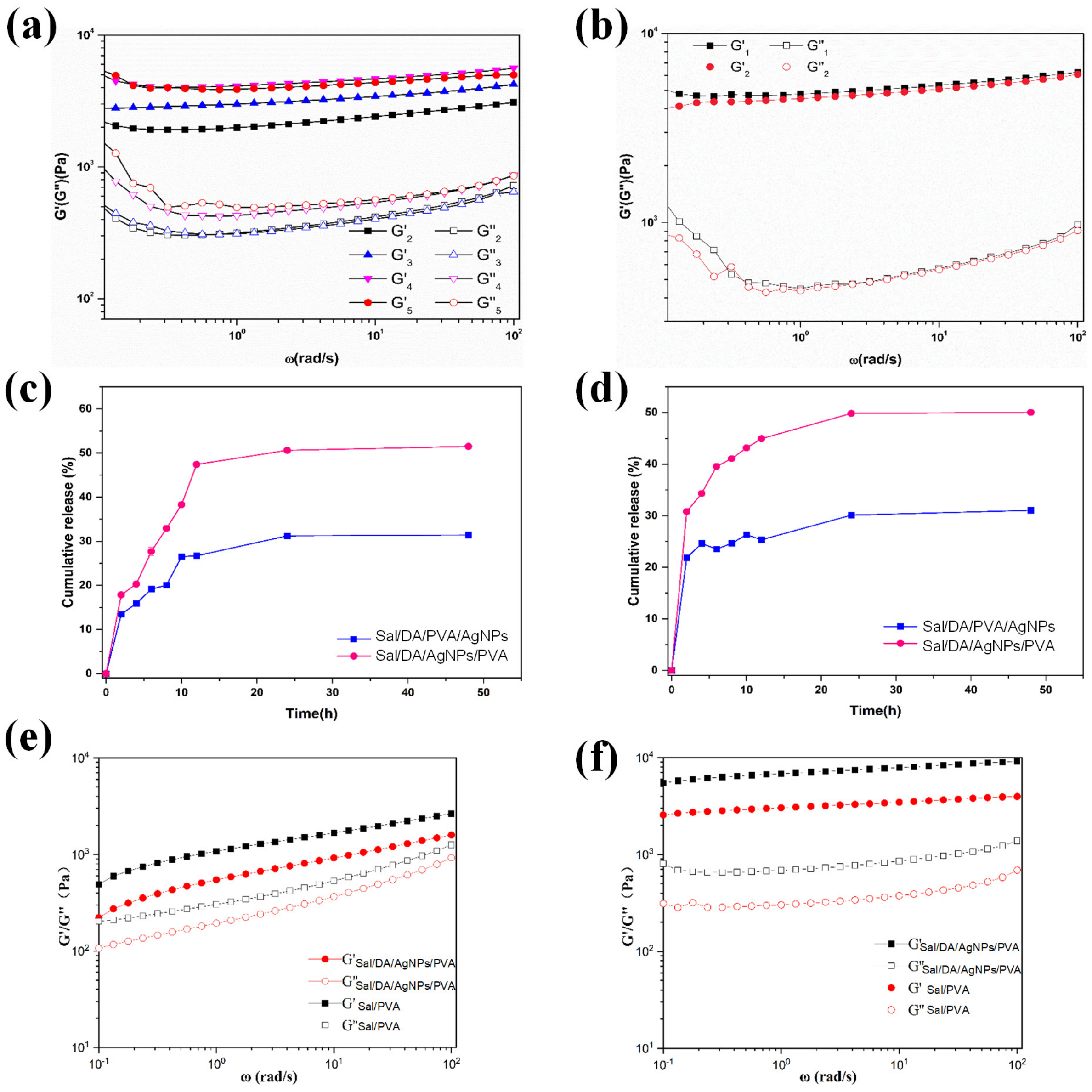

Figure S1. (a) Oscillatory frequency sweep curves of Sal/DA/AgNPs/PVA hydrogels with different number of freeze-thaw cycles; (b) Oscillatory frequency sweep curves under different Ag^+ addition sequences; (c-d) Ag^+ release profiles at pH 2.0 (c) and pH 5.4 (d); (e-f) Oscillatory frequency sweep curves of Sal/PVA and Sal/DA/AgNPs/PVA before (e) and after (f) freeze-thaw cycling.

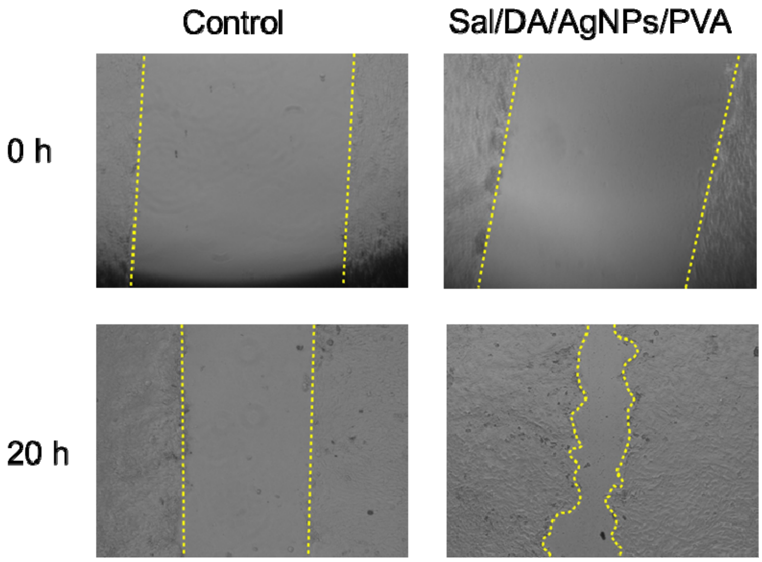

Figure S2. *In vitro* scratch assays to evaluate the effect of the Sal/DA/AgNPs/PVA composite hydrogel on cell migration.
